# Association between bilirubin levels with incidence and prognosis of stroke: A meta-analysis

**DOI:** 10.3389/fnins.2023.1122235

**Published:** 2023-02-14

**Authors:** Kun Zhao, Rui Wang, Rongrong Chen, Jialei Liu, Qing Ye, Kai Wang, Juebao Li

**Affiliations:** ^1^Department of Rehabilitation Medicine, Center for Rehabilitation Medicine, Rehabilitation & Sports Medicine Research Institute of Zhejiang Province, Zhejiang Provincial People’s Hospital, Affiliated People’s Hospital, Hangzhou Medical College, Hangzhou, Zhejiang, China; ^2^Zhejiang University School of Medicine, Hangzhou, Zhejiang, China; ^3^Bengbu Medical College, Bengbu, Anhui, China; ^4^Department of Neurology, The Second Affiliated Hospital of Xuzhou Medical University, Xuzhou, Jiangsu, China

**Keywords:** direct bilirubin, total bilirubin, ischemic stroke, stroke, meta-analysis

## Abstract

**Objective:**

Bilirubin has anti-inflammatory, antioxidant, and neuroprotective properties, but the association between bilirubin and stroke remains contentious. A meta-analysis of extensive observational studies on the relationship was conducted.

**Methods:**

Studies published before August 2022 were searched in PubMed, EMBASE, and Cochrane Library. Cohort, cross-sectional and case-control studies that examined the association between circulating bilirubin and stroke were included. The primary outcome included the incidence of stroke and bilirubin quantitative expression level between stroke and control, and the secondary outcome was stroke severity. All pooled outcome measures were determined using random-effects models. The meta-analysis, subgroup analysis, and sensitivity analysis were performed using Stata 17.

**Results:**

A total of 17 studies were included. Patients with stroke had a lower total bilirubin level (mean difference = −1.33 μmol/L, 95% CI: −2.12 to −0.53, *P* < 0.001). Compared with the lowest bilirubin level, total odds ratio (OR) of the highest bilirubin for the occurrence of stroke was 0.71 (95% CI: 0.61–0.82) and ischemic stroke was 0.72 (95% CI: 0.57–0.91), especially in cohort studies with accepted heterogeneity (*I*^2^ = 0). Serum total and direct bilirubin levels were significantly and positively associated with stroke severity. A stratified analysis based on gender showed that the total bilirubin level in males correlated with ischemic stroke or stroke, which was not noted in females.

**Conclusion:**

While our findings suggest associations between bilirubin levels and stroke risk, existing evidence is insufficient to establish a definitive association. Better-designed prospective cohort studies should further clarify pertinent questions (PROSPERO registration number: CRD42022374893).

## Introduction

Stroke is a clinical syndrome of brain injury due to blood vessel blockage or blood vessel rupture and bleeding classified as ischemic and hemorrhagic stroke (Campbell and Khatri, 2020). Intravenous plasminogen activator is currently the gold standard treatment for patients with acute ischemic stroke. However, it is prone to cause adverse reactions such as cerebral hemorrhage, and most patients arriving at the stroke center exceed the optimal treatment window of 4.5 h (Thakkar et al., 2019). Therefore, early prediction of stroke occurrence and identification of stroke prognostic factors are important for stroke management.

Bilirubin, an end-product of heme catabolic pathway, has long been regarded as a potentially toxic substance whose elevated levels can cause irreversible damage to the brain and nervous system. However, there is strong evidence that bilirubin has anti-inflammatory, antioxidant, and neuroprotective properties (Thakkar et al., 2019). Previous meta-analyses suggested that a higher total level of bilirubin was related to a lower stroke prevalence and had a significant association with stroke severity, indicating that bilirubin might be involved in the progression of stroke (Zhong et al., 2019; Song et al., 2022). Recently, however, there has been no definitive consensus on the relationship of bilirubin levels with stroke risk (Page et al., 2021). In addition, few studies have concentrated on direct or indirect bilirubin, limiting the explanation of the relation between stroke and bilirubin of various types. Therefore, this study aimed to systematically update the meta-analysis to investigate the connection between the different bilirubin subtypes and the risk as well as the prognostic outcome of stroke.

## Materials and methods

This study was registered on the PROSPERO (register number: CRD42022374893) and was conducted following the Preferred Reporting Items for Systematic review and Meta-analyses (PRISMA) guidelines (Page et al., 2021).

### Search strategy

The electronic databases of PubMed, EMBASE, and Cochrane Central were searched till 25 August 2022, using a string of keywords that are related to bilirubin (such as “bilirubin” or “BIL”) and stroke (such as “stroke,” “cerebral infarction”). No language restrictions have been established within the research strategy. The search strategy in detail was shown in Supplementary Table 1. To avoid the absence of documentation, manual searches were also carried out from the reference lists of all included articles and previous meta-analyses.

### Selection criteria

Inclusion criteria for included studies are as follow: (1) observational studies, including cohort studies, case-control studies, and cross-sectional studies; (2) the relationship between circulating total bilirubin level, direct bilirubin level, or indirect bilirubin level and stroke risk was investigated; and (3) reporting hazard ratio (HR), relative risk (RR), or odds ratio (OR) with the corresponding confidence intervals (CI) for stroke risk or other poor clinical outcomes, or reporting mean differences of concentrations for various bilirubin subtype between stroke patients and control group at discharge or follow-up. Exclusion criteria are as follows: (1) the study did not provide complete effect estimates or data were not available, including conference abstract, data cannot be transformed to standard format or blending stroke data with other cardiovascular diseases, and (2) articles not published in English. Screening of relevant articles to identify eligible studies for inclusion was performed separately by two investigators, and disagreements were resolved through discussion.

### Data extraction

The primary outcome was the occurrence risk of ischemic stroke or all stroke subtypes. The secondary outcome was the occurrence of poor outcomes [modified Rankin score (mRS) >2, National Institutes of Health Stroke Scale (NIHSS) ≥8] and the mean difference in bilirubin levels between patients with or without stroke. Two researchers independently reviewed the full text of potential included studies to extract pertinent information as follow: first author, location, age, gender, sample size, cases size and definition criteria, study design, sample nature, source, category of exposure (total, direct, or indirect bilirubin) and determination method, adjusted confounding factors, and outcome with summary statistics. The disagreements in this process were reviewed by discussion.

### Quality assessment

The Newcastle-Ottawa Scale (NOS) criteria was used to perform quality assessment for cohort studies and case-control studies (Wells et al., 2022). For cross-sectional studies, quality assessment using the Agency for Healthcare Research and Quality (AHRQ) criteria (Rostom et al., 2004). Disagreements about methodological quality were addressed through discussion and mutual consultation. Overall, scores of six or more were rated as being of good quality.

### Statistical analysis

The impact of bilirubin subtypes levels on all stroke or ischemic stroke risk was assessed using OR with corresponding 95% CI, and HR or RR was directly converted into OR. Because statistical results were reported in distinct ways among different studies (OR per quartile, per quintile or per 1-unit increment in the continuous bilirubin traits), the results were first transformed into the OR between the highest and lowest levels (reference group) for each study. Individual adjusted OR and 95% CI were preferentially estimated for pooling. For studies that investigated the difference in bilirubin subtype levels between stroke patients and the control group, we pooled the mean differences by meta-analysis. All units were converted to μmol/L and 1 mg/L total bilirubin equal to 17.1 μmol/L if not consistent.

Heterogeneity between the studies was estimated using the Cochran *Q* test and statistical method *I*^2^, and low, moderate and high levels of heterogeneity were cut-off with the values of 25, 50, and 75%, respectively (Higgins et al., 2003). Due to the variation in study characteristics, we assumed that the actual effect size may vary from study to study as the existence of clinical heterogeneity, random-effects model of DerSimonian and Laird method were determined to perform meta-analysis (DerSimonian and Laird, 1986). Pre-established subgroup analyses of study design, gender, and bilirubin subtypes were conducted. Sensitivity analysis was conducted by omitting one study by turns to examine the robustness of pooled risk estimates. If the number of studies included for the outcome indicators exceeds 10, the funnel plot and egger test are used for qualitative and quantitative testing of publication bias. Statistical analyses were performed using STATA version 17.0. Two-sided *P*-values below 0.05 were considered statistically significant.

### Certainty of evidence assessment

The Grading of Recommendations Assessment, Development and Evaluation (GRADE) approach which graded the evidence as “high,” “moderate,” “low,” or “very low” was used to assess the certainty of the evidence (Guyatt et al., 2011).

## Results

### Study identification and selection

**FIGURE 1:**
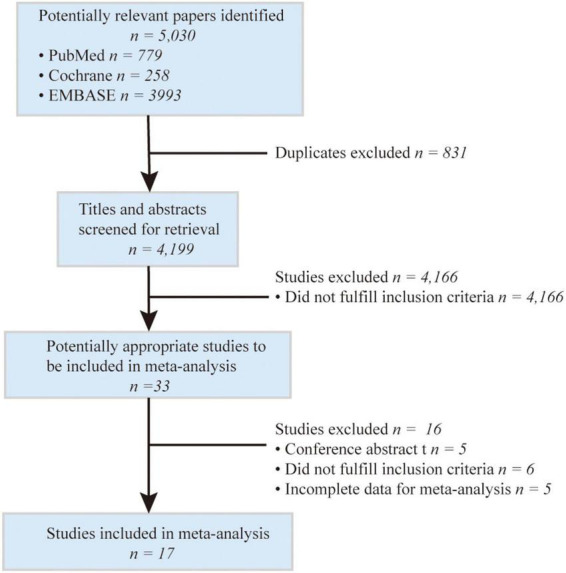
PRISMA Flow chart of the study collection for the present review and meta-analysis.

The searching and screening process of the included studies are shown in Figure 1. The initial search included 5,030 potential studies from databases (PubMed, EMBASE, and Cochrane Library). After the exclusion of duplicates and irrelevant studies, 33 reports were left for retrieval. After the full-text search, 17 studies were included in the final meta-analysis (Perlstein et al., 2008; Pineda et al., 2008; Kimm et al., 2009; Ekblom et al., 2010; Luo et al., 2012; Oda and Kawai, 2012; Xu et al., 2013; Jørgensen et al., 2014; Li et al., 2014, 2020; Mahabadi et al., 2014; Zhou et al., 2014; Kunutsor et al., 2015; Lee et al., 2017; Marconi et al., 2018; Liu et al., 2020; Peng et al., 2022; Figure 1).

### Study characteristics

Table 1 lists study-level characteristics and shows the quality assessment score of included studies. Seventeen studies between 2008 and 2022 were included, including 7 prospective cohort studies (Kimm et al., 2009; Ekblom et al., 2010; Luo et al., 2012; Mahabadi et al., 2014; Kunutsor et al., 2015; Lee et al., 2017; Marconi et al., 2018), and 10 cross-sectional studies (Perlstein et al., 2008; Pineda et al., 2008; Oda and Kawai, 2012; Xu et al., 2013; Jørgensen et al., 2014; Li et al., 2014, 2020; Zhou et al., 2014; Liu et al., 2020; Peng et al., 2022). The sample size varied between 316 and 96,381, involving a total of 12,081 stroke patients. Three studies presented their respective findings for men and women (Kimm et al., 2009; Ekblom et al., 2010; Oda and Kawai, 2012). Based on the NOS criteria, all studies are of good quality.

**TABLE 1:** The characteristic of included studies.

References	Country	Design	Sample size	Age (years)	Male (%)	Cases	Category of exposure	Exposure level	Adjustment	Outcome	Study quality (NOS/AHRQ)
Perlstein et al., 2008	USA	Cross-sectional	13,214	≥20	48.1	453, AS	Total bilirubin	Highest: 0.8–12.9 Lowest: 0.1–0.5	Age, sex, race/ethnicity, smoking, hypertension, total to HDL cholesterol ratio, and diabetes	Stroke prevalence and adverse stroke outcomes	7
Pineda et al., 2008	USA	Cross-sectional	743	67.5 ± 16.6	47.60	743	Direct (Dbil) bilirubin	Highest: ≤0.1 mg/dl Lowest: ≥0.4 mg/dl	Sex, serum glucose, prior antithrombotic use, hypertension, atrial fibrillation, and mRS scores	Adverse stroke outcomes (NIHSS >12)	9
Kimm et al., 2009	Korea	Prospective cohort	78,724	30–89	52.15	1,964 1,189 473	Total bilirubin	Highest: 22.2–34.2 Lowest: 0–10.2	Age, smoking (nonsmoker, ex-smoker, and current smoker), alcohol (yes or no), exercise (yes or no), ALT, GGT, total cholesterol, type 2 diabetes, and hypertension	Stroke prevalence	9
Ekblom et al., 2010	Sweden	Prospective cohort	693	25–74	55	231	Total bilirubin	Unclear	Age, BMI, systolic blood pressure, smoking, apolipoprotein B/A1, diabetes and hsCRP	Stroke prevalence	7
Luo et al., 2012	China	Prospective cohort	531	67.00 ± 12.9	63.5	531	Total bilirubin Direct (Dbil) bilirubin	Highest: ≥22.2 Lowest: 0–10.2 Highest: ≥6.84 Lowest: 0–3.42	BG, TC, HDL-C, hypertension, AF, sex, and age	Adverse stroke outcomes (NIHSS ≥8)	9
Oda and Kawai, 2012	Japan	Cross-sectional	5,444	>18	62	92	Total bilirubin	Highest: 17.9–71.8 Lowest: 2.6–9.3	Age, aspartate aminotransferase, alanine aminotransferase, γ-glutamyl transferase, current smoking, physical activity, and everyday drinking	Stroke prevalence	6
Xu et al., 2013	China	Cross-sectional	2,361	NS	63.2	2,361	Total bilirubin Direct bilirubin	Highest: 18.0–88.0 Lowest: 1.0–10.0 Highest: 4.2–37 Lowest: 0.4–2.0	Age, sex, alcohol consumption, cigarette smoking, blood levels of glucose and lipids, admission SBP and DBP, blood urea nitrogen, serum creatinine, sodium, hematocrit, history of stroke, hypertension, diabetes, coronary heart disease, rheumatic heart disease, and atrial fibrillation, family history of stroke, hypertension, and diabetes	Adverse stroke outcomes (NIHSS ≥10)	8
Jørgensen et al., 2014	16 countries	Cross-sectional	9,742	≥55	57.4	221	Total bilirubin	Highest: >13 Lowest: ≤8	Sex, age, and sibutramine/placebo	Stroke prevalence	8
Li et al., 2014	China	Cross-sectional	2,856	30–69	63.9	343	Total bilirubin	Highest: >13.9 Lowest: ≤7.8	Sex, BMI, smoking status, DBP, LDL-C, FPG, eGFR, TB, DM, and baPWV	Stroke prevalence	8
Mahabadi et al., 2014	Germany	Prospective cohort	3,553	45–75	44	95	Total bilirubin	/	Age, gender, BMI, systolic blood pressure, LDL, HDL, antihypertensive medication, lipid-lowering medication, diabetes, smoking status, and CAC score	Stroke prevalence	9
Zhou et al., 2014	China	Cross-sectional	1,098	>18	45.7	733	Total bilirubin	Highest: >12.99 Lowest: ≤9.58	Age, sex, and vascular risk factors	Stroke prevalence	8
Kunutsor et al., 2015	Netherlands	Prospective cohort	7,222	28–75	48.5	159	Total bilirubin	Per 1-SD higher	Age and sex, smoking status, history of diabetes, systolic blood pressure, total cholesterol, high-density lipoprotein cholesterol, and BMI, alcohol consumption, glucose, and triglycerides, γ-glutamyl transferase, and alanine aminotransferase	Stroke prevalence	9
Lee et al., 2017	Korea	Prospective cohort	5,599	>18	66.9	806	Total bilirubin		Age, sex, systolic blood pressure, fasting serum glucose, total cholesterol, high-density lipoprotein-cholesterol, and smoking status	Stroke prevalence	9
Marconi et al., 2018	USA	Prospective cohort	96,381	48 (mean)	97	2,112	Total bilirubin	Highest: ≥15.39 Lowest: ≤6.84	Age, sex, race-ethnicity, systolic blood pressure, smoking, diabetes mellitus, total cholesterol, high-density lipoprotein cholesterol, HIV, hepatitis C, liver fibrosis measured by FIB-4, alcohol abuse/dependence, cocaine, and obesity	Stroke prevalence	9
Li et al., 2020	China	Cross-sectional	610	66.7 (mean)	63.11	610	Total bilirubin	/	High density lipoprotein cholesterol, and triglyceride	Adverse stroke outcomes	9
Liu et al., 2020	China	Cross-sectional	316	70.36 ± 10.06	42.72	42	Total bilirubin	Highest: 34.15 ± 9.78 Lowest: ≤8.04 ± 2.03	Age, sex, body mass index, systolic blood pressure, the congestive heart failure, hypertension, age >75 years, diabetes, and previous stroke/transient ischemic attack score, left ventricular ejection fraction, left atrial diameter, alanine aminotransferase, aspartate aminotransferase, uric acid, total cholesterol, low-density lipoprotein cholesterol, high-density lipoprotein cholesterol, triglycerides, estimated glomerular filtration rate, heart failure, coronary artery disease, hypertension, diabetes, drinking, smoking, international standardized ratio value, and taking oral anticoagulant and antiplatelet drugs	Stroke prevalence	9
Peng et al., 2022	China	Cross-sectional	585	64.9 ± 12.2	66.5	585	Total bilirubin Direct bilirubin Indirect bilirubin		Age, sex, onset-time to treatment, admission glucose, admission ALT, admission AST, current smoking, alcohol drinking, history of stroke, cerebral hemorrhage, hypertension, diabetes mellitus, and hyperlipidemia, admission NIHSS score	Adverse stroke outcomes	8

### Primary outcome

**FIGURE 2:**
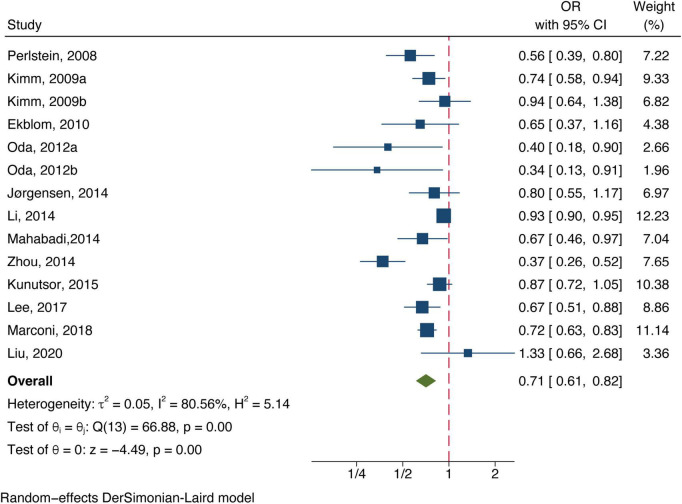
Forest plot of the association between total bilirubin level and stroke prevalence.

**FIGURE 3:**
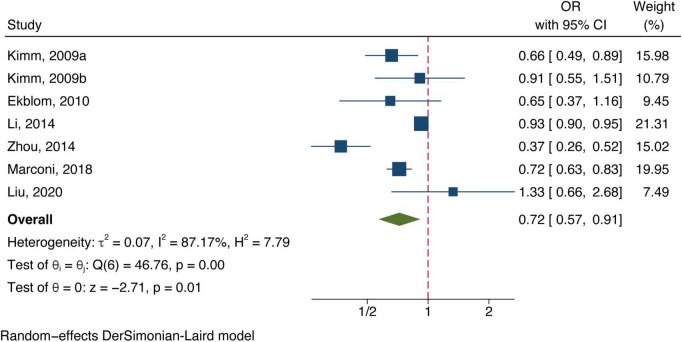
Forest plot of the association between total bilirubin level and ischemic stroke prevalence.

Twelve included studies examined the association of total bilirubin level with stroke, Figure 2 shows the adjusted ORs for each study and the pooled OR with the highest vs. lowest bilirubin level groups. Heterogeneity (*I*^2^ = 80.56%, *P* < 0.01) was observed and compared to the group at the lowest bilirubin level group, the risk of stroke was significantly lower among participants at the highest bilirubin level (OR = 0.71, 95% CI: 0.61–0.82, *P* < 0.01). Similarly, the random-effect model analysis comparing ischemic risk and total bilirubin level quartiles showed a significant inverse association, with a pooled effect OR of 0.72 (95% CI: 0.57–0.91, *P* = 0.01, Figure 3) and a high degree of heterogeneity (*I*^2^ = 87.17%, *P* < 0.01). Sensitivity analysis revealed no apparent influence of an individual study on the results of meta-analysis for all types of stroke (Supplementary Figure 1). However, when the Marconi et al. (2018) study was excluded, the findings did not reveal any significant association with the risk of ischemic stroke (OR = 0.73, 95% CI: 0.51–1.03, *P* = 0.07), implying that the pooled risk estimates were not robust (Supplementary Figure 2). No studies were conducted on the relationship between direct or indirect bilirubin levels and the risk of stroke or ischemic stroke.

### Secondary outcome

Nine studies reported the differences in different bilirubin types level between stroke patients and the control group. Six studies showed that the group of stroke patients had a significantly lower total bilirubin level (mean difference = −1.33 μmol/L, 95% CI: −2.12 to −0.53, *P* < 0.01, Supplementary Figure 3). Sensitivity analysis by omitting one study each time also revealed no evident influence on the pooled results (Supplementary Figure 4). Direct bilirubin and indirect bilirubin level did not show a significant difference due to the limited included studies (Supplementary Figure 3).

**FIGURE 4:**
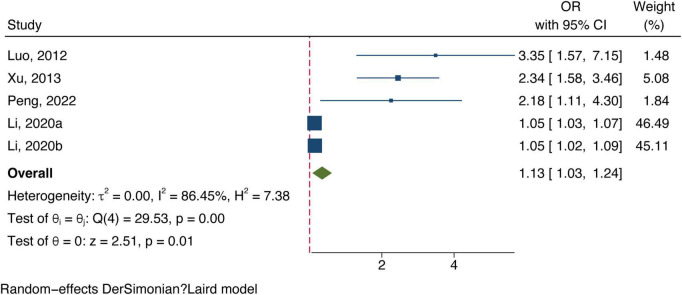
Forest plot of the association between total bilirubin level and ischemic stroke severity.

**FIGURE 5:**
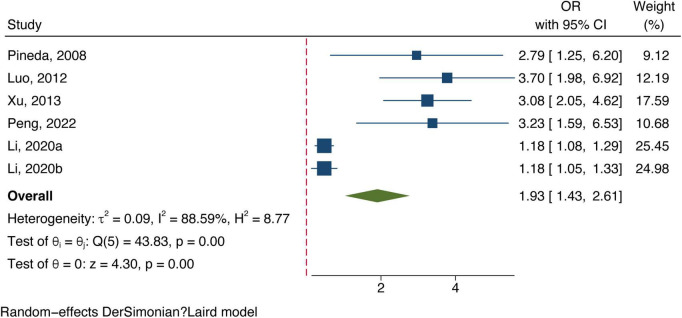
Forest plot of the association between direct bilirubin level and ischemic stroke severity.

All included studies concentrated on associating bilirubin subtypes with ischemic stroke severity. Five data groups were obtained from four studies examining total bilirubin levels and the severity of ischemic stroke. [Three studies (Luo et al., 2012; Xu et al., 2013; Li et al., 2020) defined severe strokes as NIHSS score ≥8 and one study defined them as 3–6 in mRS (Peng et al., 2022).] The meta-analysis results showed a positive correlation between total bilirubin level and stroke poor outcome (OR: 1.13, 95% CI: 1.03–1.24) with a high degree of heterogeneity (*I*^2^ = 86.45%) (shown in Figure 4). Six datasets of direct bilirubin from five studies were pooled, and the findings indicated that direct bilirubin level was also positively associated with the poor outcome of ischemic stroke (OR: 1.93, 95% CI: 1.43–2.61, and *I*^2^ = 88.59%) (shown in Figure 5). Additionally, when we performed sensitivity analyses, the meta-analyses results were robust (Supplementary Figures 5, 6). Not enough data was collected because we could not pool the correlation between indirect bilirubin level and ischemic stroke severity.

### Subgroup analysis

For the primary outcome, prespecified subgroup analyses by study design, gender, and location were performed (Table 2). The results showed less heterogeneity after being grouped by study design. No substantial heterogeneity was found among prospective cohorts (*I*^2^ = 0.00%) and pooled results for a significant relationship between stroke (OR: 0.75, 95% CI: 0.69–0.82) and ischemic stroke patients (OR: 0.72, 95% CI: 0.64–0.81). Three studies separately reviewed the relationship between total bilirubin level and stroke risk in males and females, respectively. Two of them examined the correlation between total bilirubin level and the risk of ischemic stroke. Interestingly, we observed a statistical association between a low risk of stroke or ischemic stroke with higher total bilirubin levels in the male population, but this statistical difference disappeared in females.

**TABLE 2:** Subgroup analysis of the correlation between bilirubin level and all stroke or ischemic stroke risk.

Subgroups	Stroke risk			Ischemic stroke risk		
	Number of groups of data	Pooled OR [95% CI]	Heterogeneity	Number of groups of data	Pooled OR [95% CI]	Heterogeneity
**Study design**						
Cohort	7	0.75 [0.69, 0.82]	*I*^2^ = 0%, *P* = 0.49	4	0.72 [0.64, 0.81]	*I*^2^ = 0%, *P* = 0.74
Cross-sectional	7	0.63 [0.43, 0.91]	*I*^2^ = 86.74%, *P* < 0.01	3	0.74 [0.37, 1.49]	*I*^2^ = 93.27%, *P* < 0.01
**Gender**						
Male	3	0.71 [0.55, 0.91]	*I*^2^ = 6.34%, *P* = 0.34	2	0.68 [0.52, 0.90]	*I*^2^ = 0%, *P* = 0.61
Female	3	0.55 [0.25, 1.18]	*I*^2^ = 65.87%, *P* = 0.05	2	0.64 [0.26, 1.54]	*I*^2^ = 61.61%, *P* = 0.11

OR, odds ratio; CI, confidence intervals.

### Publication bias

We tested the publication bias for the outcome of the correlation between total bilirubin level and stroke risk, and the *P*-values of the egger test were 0.08, indicating no inflation of effect sizes due to selective publication. The funnel plot was performed in Supplementary Figure 7.

### GRADE assessment

The Supplementary Table 2 shows that the level of the evidence was graded as “low” owing to upgrade for dose-response and downgrades for inconsistency.

## Discussion

To more comprehensively and accurately assess the influence of bilirubin subtype levels on stroke outcomes, we performed this comprehensive updated meta-analysis. The pooled results showed that bilirubin level was negatively associated with ischemic stroke and stroke risk after adjustment. Furthermore, a highly significant relationship was detected between total bilirubin levels or direct bilirubin levels and ischemic stroke severity. The sensitivity analysis provided additional insight into the robustness of the pooled risk estimates. Moreover, subgroup analyses by gender showed a statistical association between a low risk of stroke or ischemic stroke with higher total bilirubin levels in the male population, but this statistical difference disappeared in females. As a result, this meta-analysis confirms that bilirubin levels at the onset are a biomarker for improved diagnosis and prognosis in stroke patients.

The relationship between total bilirubin level and all types of stroke or ischemic stroke risk was consistent with previous studies (Zhong et al., 2019). Unlike it, we excluded Ren et al.’s (2016) study, because its outcome was cardiovascular disease, including prior coronary heart disease or peripheral arterial disease with stroke. However, it did not provide data on stroke separately, which will lead to biased results. In addition, we have added two new studies (Marconi et al., 2018; Liu et al., 2020) to confirm the link between bilirubin levels and stroke more comprehensively and accurately. It was worth mentioning that Liu’s study reported that each 1 mmol/L increase in total bilirubin would increase the risk of first ischemic stroke in patients with non-valvular atrial fibrillation. The sensitivity analysis showed that this did not affect the robustness of the conclusion. Our meta-analysis observed a negative statistical association between ischemic stroke/stroke and total bilirubin level in the male population, but this statistical difference disappeared in females. These results were consistent with the study of Zhong and his colleagues (Zhong et al., 2019). The difference in bilirubin levels between gender might be attributed to differences in heme oxygenase, serum oestrogen, iron storage, and the influence of lifestyle (Zhong et al., 2019). Our subgroup analysis of prospective cohort studies indicated that total bilirubin level was inversely associated with the incidence of all stroke or ischemic stroke with low heterogeneity. This result again emphasized that bilirubin level was essential in developing stroke risk. However, the causal relationship needs to be confirmed by further cohort studies, as genetic evidence according to Mendelian randomization approaches did not suggest any causal effect of bilirubin levels on developing stroke in Koreans (Lee et al., 2017). Therefore, Mendelian randomization studies based on different races should also be carried out in the future to avoid ignoring some important information or leading to erroneous conclusions. In addition to diagnostic value, we investigated the prognostic value of bilirubin subtypes in ischemic stroke. Our results determined that the direct and total bilirubin levels were positively correlated with the ischemic stroke severity, consistent with the systematic review of Ghojazadeh et al.’s (2020) study and Song et al.’s (2022) study. Song et al. also established that bilirubin is linear with ischemic stroke severity and trial sequential analysis found that the sample size for this study is sufficient. A cross-sectional descriptive analysis conducted by Sagheb Asl et al. (2018) also showed that total, direct and indirect bilirubin levels was significantly associated with mortality in ischemic stroke patients. Total bilirubin and direct bilirubin may be critical endogenous antioxidants and their levels can reflect the stroke severity and can be used as an auxiliary indicator. Because of the limited number of studies, future investigations may explore the association between indirect bilirubin levels and stroke severity.

Ischemic stroke is characterized by a sudden loss of blood circulation in a focal area of the brain, preventing the proper delivery of glucose, oxygen, and nutrients, causing chemokines, cytokines, and reactive oxygen species (ROS) trigger inflammatory responses (Maida et al., 2020). ROS can stimulate many signal transduction pathways important for maintaining neuronal homeostasis, but the overproduction of ROS can induce structural and functional damage of neurons throughout the whole process of acute ischemic stroke, leading to brain injury (Niizuma et al., 2009). Bilirubin is an endogenous antioxidant that protects against the oxidation of low-density lipoprotein cholesterol, scavenging oxygen-free radicals boosting heme oxygenase activity and helping serum cholesterol to dissolve (Schwertner et al., 1994). Bilirubin contains an extended conjugated double bond system and a reactive hydrogen atom, which has strong antioxidant properties, thus preventing the generation of cellular ROS and the formation of atherosclerotic plaques (Vogel et al., 2017), thereby avoiding the onset of stroke; at the same time bilirubin can affect the inflammatory pathways by preventing the connection between C1q and immunoglobulins, significantly reduces the ability of complement to initiate through the traditional pathway, controlling the proliferation of T-regulatory cells (Tregs), modifies the activity of cytotoxic T-lymphocytes, blocking the production of pro-inflammatory cytokines, as the recruitment of pro-inflammatory cytokines is one of important factors in the formation of stroke (Thakkar et al., 2019); it can also resist myeloperoxidase-induced protein or lipid oxidation, scavenges hypochlorous acid, and prevents stroke (Boon et al., 2015). The exact mechanism linking direct bilirubin level to the high incidence of ischemic stroke remains unclear, and some possible explanations can be suggested. Direct bilirubin is more soluble in serum than the lipophilic indirect bilirubin, thus making direct bilirubin an active form that is more readily available than indirect bilirubin (Hansen et al., 2020). Additionally, as a systemic disease, an elevated level of direct bilirubin may indicate the injury of hepatocytes (Sharma et al., 2021); therefore, the positive association of direct bilirubin levels with poor clinical outcomes might be due to the hepatic dysfunction. Future studies are required to demonstrate the specific differences in various bilirubin subtypes concerning their molecular mechanisms of action.

In summary, the relationship between bilirubin levels and stroke is complex. On the one hand, the production of bilirubin was physiologically enhanced in response to oxidative stress. When bilirubin level was upregulated through the stroke, we suppose it would play an important role. Additionally, strokes with higher severity are accompanied by higher levels of oxidative stress, which may also induce elevated anti-oxidative power reflected by the level of bilirubin (Domínguez et al., 2010). On the other hand, upregulated bilirubin can protect neurons against oxidation between a specific concentration range. At pathologic levels, bilirubin has been considered as a neurotoxic agent. Therefore, future studies can explore the impact of high-concentration bilirubin levels on the nervous system of stroke, so as to provide more decision-making suggestions for drug use.

This study is a meta-analysis of observational studies investigating the association between bilirubin levels of various subtypes and strokes from the most comprehensive literature research for now. The enrolled studies were analyzed according to the adjusted results. The limitations of this study also need to be recognized. Firstly, the substantial heterogeneity of included studies suggests that applicability of the results should be interpreted with caution. Secondly, the sample nature and the laboratory measurement method of bilirubin level were inconsistent, which may lead to some bias into the results. Thirdly, only a few studies in this meta-analysis have taken gender into consideration. As previous studies have revealed, the association between the bilirubin level and stroke risk was only confirmed in the males population using a gender stratified analysis, there is a lot of space remained to be explored in these results (Zhong et al., 2019). Fourthly, due to the limited number of studies, some outcomes have not enough power to be addressed in the clinical practice. More research is needed to investigate this relationship in the future. Lastly, hemorrhagic stroke is also a subtype of stroke, but only one showed that total bilirubin levels were not significantly associated with hemorrhagic stroke risk (Kimm et al., 2009). The relationship between bilirubin level and hemorrhagic stroke needs further investigation.

## Conclusion

Although our findings suggested a negative association between total bilirubin levels and all stroke or ischemic stroke risk and a highly significant relationship between total/direct bilirubin levels and the severity of ischemic stroke, the existing evidence is inadequate to establish a definitive conclusion. More sophisticated prospective cohort studies and further analyses are required to further explain relevant issues.

## Data availability statement

The original contributions presented in this study are included in this article/Supplementary material, further inquiries can be directed to the corresponding authors.

## Author contributions

KZ and JBL contributed to the study design and data research. RW, RC, and JLL contributed to study selection and quality evaluation. KZ, RW, and KW contributed to statistical analysis. KZ, RC, JLL, QY, KW, and JBL contributed to drafting of the manuscript and language modification. All authors contributed to the article and approved the submitted version.
