# Supplementary material for: Association between bilirubin levels with incidence and prognosis of stroke: A meta-analysis
Source: Front Neurosci. 2023 Feb 14;17:1122235. doi: 10.3389/fnins.2023.1122235 (PMC9971723; doi:10.3389/fnins.2023.1122235)
Supplement: Supplementary file 1 [file Table_1.docx]

Table S1. Search strategy

| **Database** | **#** | **Search strategy** | **Results** |
| --- | --- | --- | --- |
| PubMed | 1 | "cerebrovascular disorders"[MeSH] OR "brain injuries"[MeSH] | 480,814 |
|  | 2 | stroke*[Title/Abstract] OR CVA[Title/Abstract] OR poststroke[Title/Abstract] OR post‐stroke[Title/Abstract] | 301,957 |
|  | 3 | cerebral[Title/Abstract] OR cerebellar[Title/Abstract] OR brain*[Title/Abstract] OR vertebrobasilar[Title/Abstract] | 1,478,735 |
|  | 4 | infarct*[Title/Abstract] OR ischaemi*[Title/Abstract] OR thrombo*[Title/Abstract] OR emboli*[Title/Abstract] OR apoplexy[Title/Abstract] OR cerebrovasc*[Title/Abstract] | 881,400 |
|  | 5 | #3 AND #4 | 124,142 |
|  | 6 | cerebral[Title/Abstract] OR brain[Title/Abstract] OR subarachnoid[Title/Abstract] | 1,401,174 |
|  | 7 | haemorrhage[Title/Abstract] OR hemorrhage[Title/Abstract] OR haematoma[Title/Abstract] OR hematoma[Title/Abstract] OR bleed*[Title/Abstract] | 450,529 |
|  | 8 | #6 AND #7 | 74,653 |
|  | 9 | #1 OR #2 OR #5 OR #8 | 672,416 |
|  | 10 | bilirubin[MeSH] | 25,904 |
|  | 11 | bilirubin* [Title/Abstract] OR BIL[Title/Abstract] OR STBL [Title/Abstract] OR IBIL[Title/Abstract] OR DBIL[Title/Abstract] OR Hematoidin[Title/Abstract] | 43,911 |
|  | 12 | #10 OR #11 | 53,030 |
|  | 13 | #9 AND #12 | 779 |
| Cochrane | 1 | MeSH descriptor: [Cerebrovascular Disorders] explode all trees | 17,426 |
|  | 2 | MeSH descriptor: [Brain Injuries] explode all trees | 2,733 |
|  | 3 | #1 OR #2 | 19,922 |
|  | 4 | (stroke*):ti,ab,kw OR (CVA):ti,ab,kw OR (poststroke):ti,ab,kw OR ("post-stroke"):ti,ab,kw | 63,916 |
|  | 5 | (cerebral):ti,ab,kw OR (cerebellar):ti,ab,kw OR (brain*):ti,ab,kw OR (vertebrobasilar):ti,ab,kw | 85,138 |
|  | 6 | (infarct*):ti,ab,kw OR (ischaemi*):ti,ab,kw OR (thrombo*):ti,ab,kw OR (emboli*):ti,ab,kw OR (apoplexy):ti,ab,kw | 100,371 |
|  | 7 | #5 AND #6 | 14,210 |
|  | 8 | (cerebral):ti,ab,kw OR (brain):ti,ab,kw OR (subarachnoid):ti,ab,kw | 85,834 |
|  | 9 | (haemorrhage):ti,ab,kw OR (hemorrhage):ti,ab,kw OR (haematoma):ti,ab,kw OR (hematoma):ti,ab,kw OR (bleed*):ti,ab,kw | 73,356 |
|  | 10 | #8 AND #9 | 11,475 |
|  | 11 | #3 OR #4 OR #7 OR #10 | 80,007 |
|  | 12 | MeSH descriptor: [Bilirubin] explode all trees | 879 |
|  | 13 | (bilirubin*):ti,ab,kw OR (Hematoidin):ti,ab,kw OR (BIL):ti,ab,kw OR (STBL):ti,ab,kw OR (IBIL):ti,ab,kw OR (DBIL):ti,ab,kw | 10,629 |
|  | 14 | #12 OR #13 | 10,629 |
|  | 16 | #11 AND #14 | 258 |
| Embase | 1 | 'cerebrovascular disease'/exp OR 'brain injury'/exp | 1,013,250 |
|  | 2 | stroke*:ab,ti OR cva:ab,ti OR poststroke:ab,ti OR 'post‐stroke':ab,ti | 472,903 |
|  | 3 | cerebral:ab,ti OR cerebellar:ab,ti OR brain*:ab,ti OR vertebrobasilar:ab,ti | 1,913,418 |
|  | 4 | infarct*:ab,ti OR ischaemi*:ab,ti OR thrombo*:ab,ti OR emboli*:ab,ti OR apoplexy:ab,ti OR cerebrovasc*:ab,ti | 1,270,852 |
|  | 5 | #3 AND #4 | 176,204 |
|  | 6 | cerebral:ab,ti OR brain:ab,ti OR subarachnoid:ab,ti | 1,814,977 |
|  | 7 | haemorrhage:ab,ti OR hemorrhage:ab,ti OR haematoma:ab,ti OR hematoma:ab,ti OR bleed*:ab,ti | 671,588 |
|  | 8 | #6 AND #7 | 101,897 |
|  | 9 | #1 OR #2 OR #5 OR #8 | 1,164,453 |
|  | 10 | 'bilirubin'/exp | 105,375 |
|  | 11 | bilirubin*:ab,ti OR bil:ab,ti OR stbl:ab,ti OR ibil:ab,ti OR dbil:ab,ti OR hematoidin:ab,ti | 74,613 |
|  | 12 | #10 OR #11 | 124,342 |
|  | 13 | #9 AND #12 | 3,993 |

Search Date: August 25, 2022


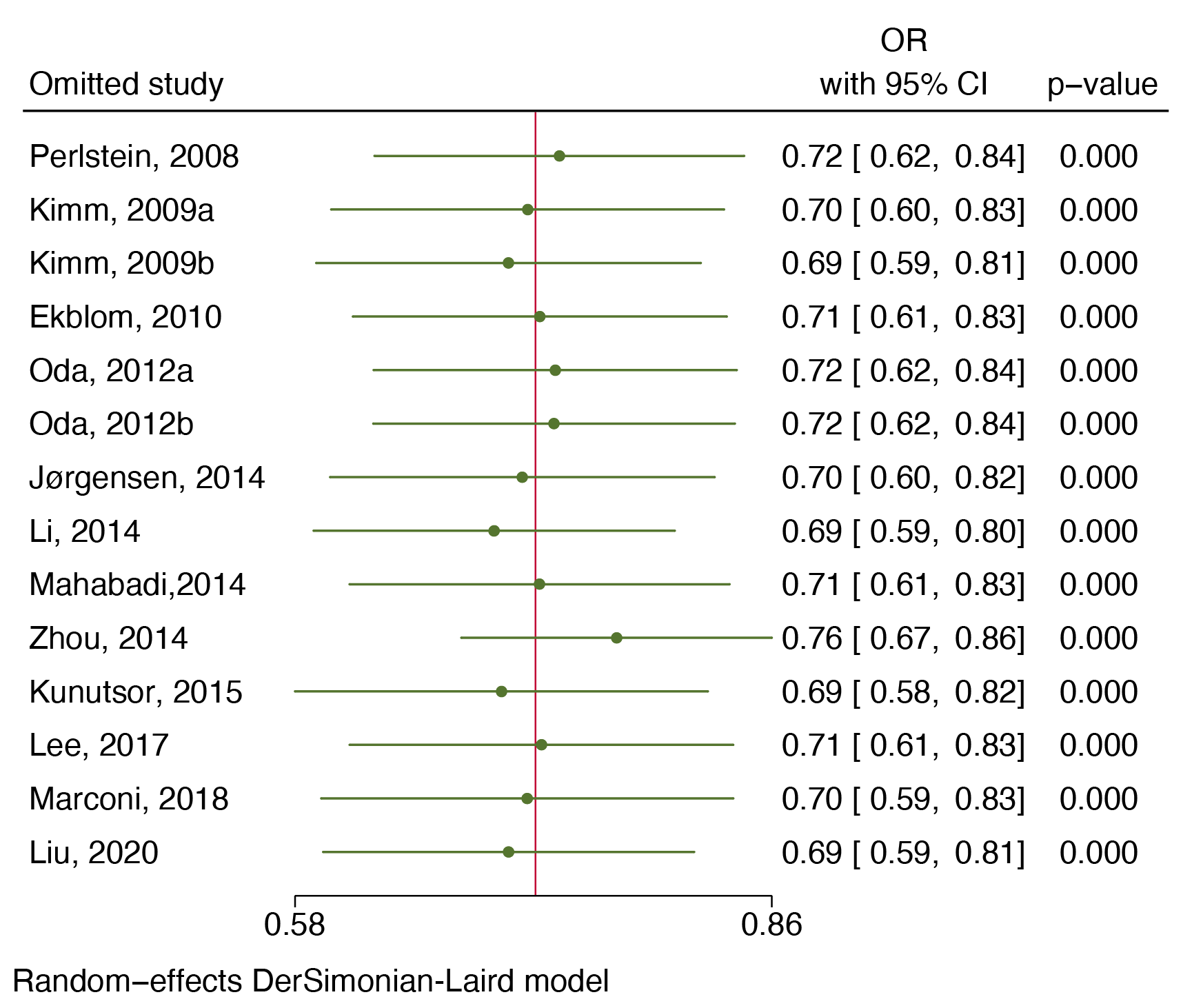


Figure S1. Forest plot in the sensitivity analysis for the association of total bilirubin level with stroke (given named study was omitted)


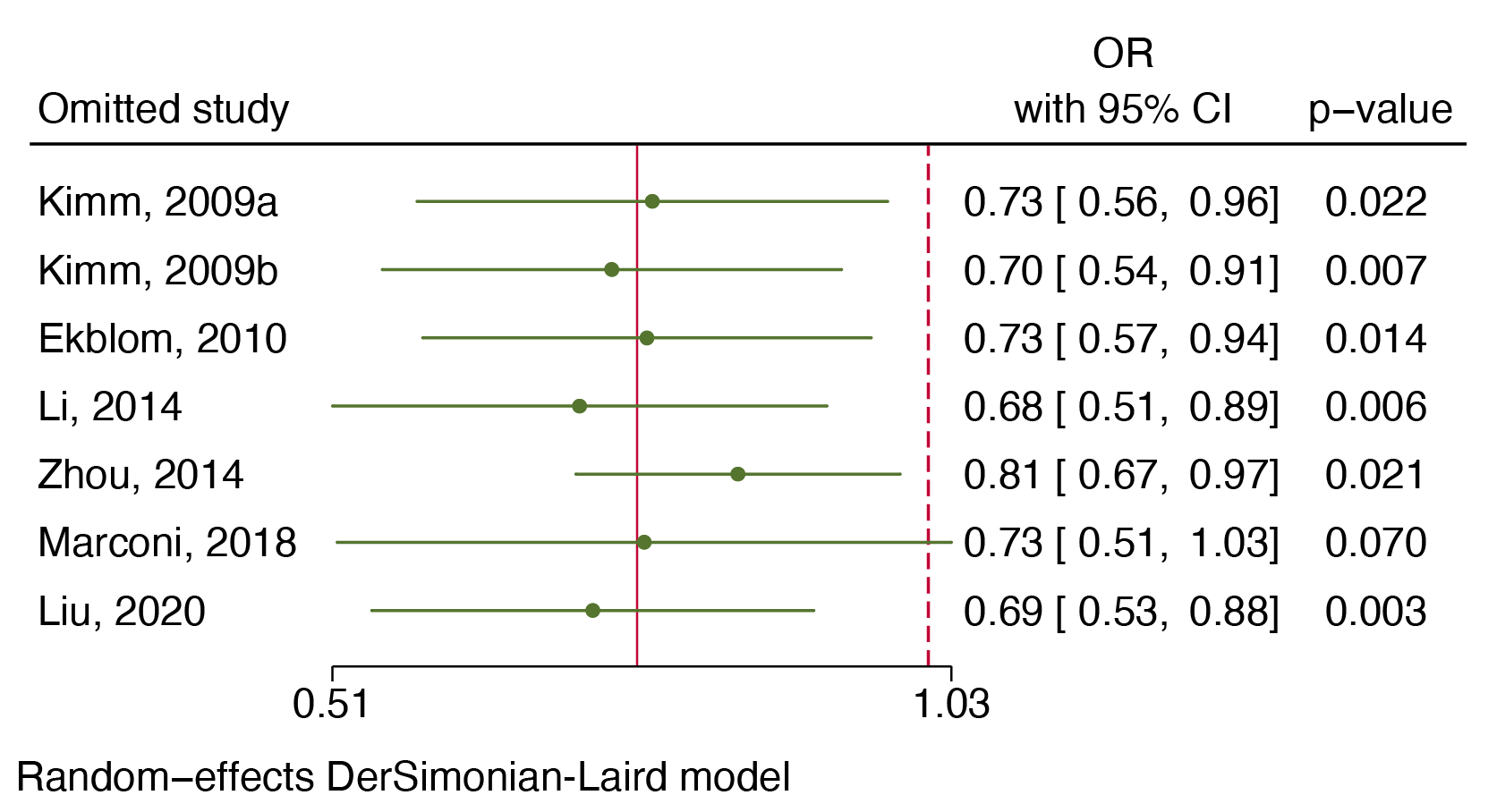


Figure S2. Forest plot in the sensitivity analysis for the association of total bilirubin level with ischemic stroke (given named study was omitted)


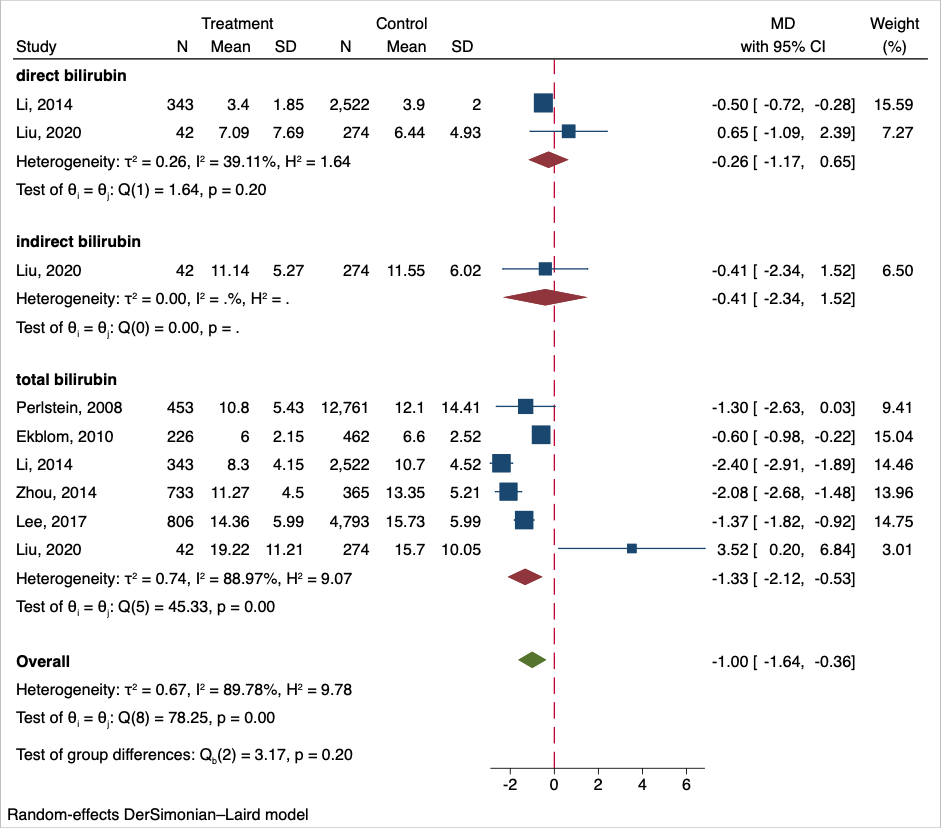


Figure S3. Mean difference of bilirubin types levels between stroke patients and the control group


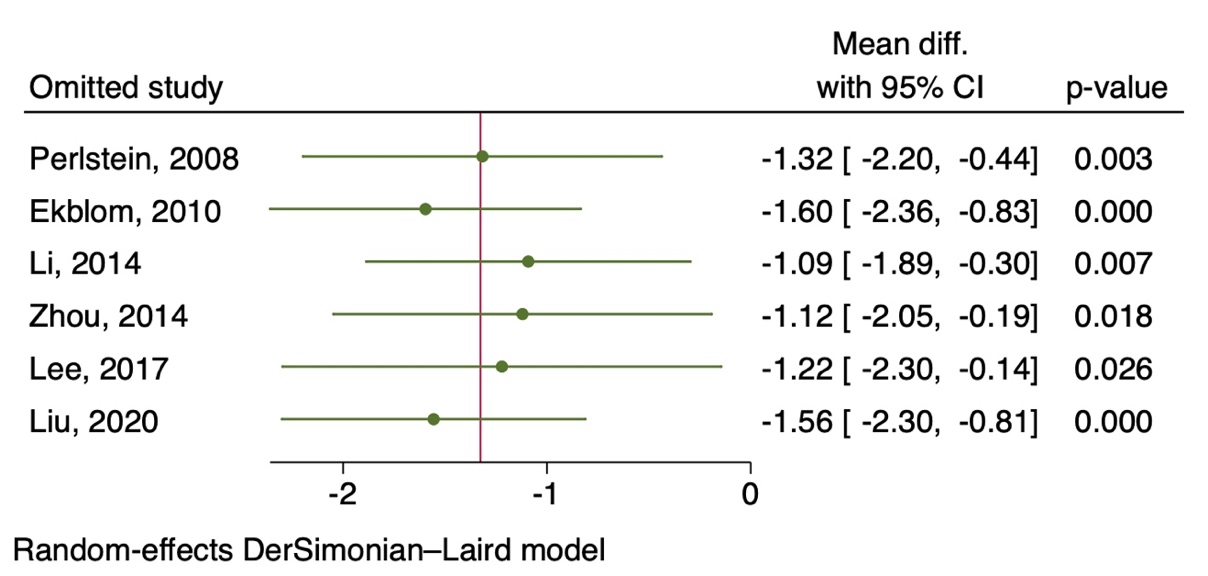


Figure S4. Forest plot in the sensitivity analysis for the differences of total bilirubin level between stroke patients and control group (given named study was omitted)

Figure S5. Forest plot in the sensitivity analysis for the association of total bilirubin level with ischemic stroke severity e (given named study was omitted)

Figure S6. Forest plot in the sensitivity analysis for the association of direct bilirubin level with ischemic stroke severity e (given named study was omitted)

Figure S7 Funnel plot to detect risk of publication bias for primary outcome

**Table S2. GRADE assessments of evidence.**

| **Category** | **No. cohort**  **comparisons** | **Downgrade quality of evidence** | | | |  | **Upgrade quality of evidence** | | | |  | **Overall quality of evidence** |
| --- | --- | --- | --- | --- | --- | --- | --- | --- | --- | --- | --- | --- |
|  |  | **Risk of bias** | **Inconsistency** | **Indirectness** | **Publication Bias** |  | **Dose–response** | **Large Effect** | **Plausible Confounding** |  | |  |
| total bilirubin level for stroke risk | 12 | not serious | serious | not serious | not serious |  | Yes | No | No |  | | ⨁⨁◯◯  Low |
| total bilirubin level for stroke severity | 5 | not serious | serious | not serious | NA |  | Yes | No | No |  | | ⨁⨁◯◯  Low |
| direct bilirubin level for ischemic stroke severity | 6 | not serious | serious | not serious | NA |  | Yes | No | No |  | | ⨁⨁◯◯  Low |

CI=confidence interval, No.=number, OR=odds ratio
